# Chemical transfer of dissolved organic matter from surface seawater to sea spray water-soluble organic aerosol in the marine atmosphere

**DOI:** 10.1038/s41598-018-32864-7

**Published:** 2018-10-05

**Authors:** Yuzo Miyazaki, Youhei Yamashita, Kaori Kawana, Eri Tachibana, Sara Kagami, Michihiro Mochida, Koji Suzuki, Jun Nishioka

**Affiliations:** 1 Institute of Low Temperature Science, Hokkaido University, Sapporo, 060-0819 Japan; 2 Faculty of Environmental Earth Science, Hokkaido University, Sapporo, 060-0810 Japan; 3 Graduate School of Environmental Studies, Nagoya University, Nagoya, 464-8601 Japan; 4 Now at School of Materials and Chemical Technology, Tokyo Institute of Technology, Tokyo, 152-8550 Japan; 5 Now at Institute for Space–Earth Environmental Research, Nagoya University, Nagoya, 464-8601 Japan

## Abstract

It is critical to understand how variations in chemical composition in surface seawater (SSW) affect the chemistry of marine atmospheric aerosols. We investigated the sea-to-air transfer of dissolved organic carbon (DOC) via cruise measurements of both ambient aerosols and SSW in the Oyashio and its coastal regions, the western subarctic Pacific during early spring. Sea spray aerosols (SSAs) were selected based on the stable carbon isotope ratio of water-soluble organic carbon (WSOC) (δ^13^C_WSOC_) and concentrations of glucose as a molecular tracer in marine aerosols together with local surface wind speed data. For both SSA and SSW samples, excitation-emission matrices were obtained to examine the transfer of fluorescent organic material. We found that the ratios of fluorescence intensity of humic-like and protein-like substances in the submicrometer SSAs were significantly larger than those in the bulk SSW (~63%). This ratio was also larger for the supermicrometer SSAs than for the SSW. The results suggest significant decomposition of protein-like DOC on a timescale of <12–24 h and/or preferential production of humic-like substances in the atmospheric aerosols regardless of the particle size. This study provides unique insights into the complex transfer of DOC from the ocean surface to the atmosphere.

## Introduction

Marine atmospheric aerosols play a key role in the climate system, as they act as cloud condensation nuclei (CCN) and ice nuclei (IN) and thus can control the atmospheric radiative budget through cloud formation^1,2^. The ocean surface is a major source of aerosols in both number and mass concentrations^3^. In particular, ocean-derived submicrometer particles contain a large fraction of water-soluble organic carbon (WSOC) as well as water-insoluble organic carbon (WIOC)^4,5^. This organic matter (OM) can significantly alter the hygroscopic property of aerosols^6–8^. -One of the largest global sources of directly emitted (primary) aerosols is wind-driven particle production at the ocean surface^4,5,9^. One important question that has remained unclear is how the production of sea spray aerosols (SSAs) with transfer of organic molecules influences the chemical properties of submicrometer SSAs at the ocean surface. In recent years, much effort has been devoted to examining linkages between the chemistry of SSAs and the biological and chemical conditions of surface seawater (SSW)^10^.

Characterization of SSAs requires sampling and analytical methods that allow isolation of these particles, preventing them from being modified by ambient gases and particles that preexist or are being transported. To assess the impact of marine biological activity on ambient aerosols, it is important to differentiate marine-derived aerosols from terrestrially (anthropogenic and plants) derived aerosols over the oceanic regions. For this purpose, some techniques need to be established to discriminate between ocean- and land-derived aerosols found in marine atmospheres. A method using the isotopic composition of aerosol carbon has been used successfully to determine the contributions of marine and terrestrial sources to aerosols found in the remote marine atmosphere^11–15^. In particular, WSOC-specific stable carbon isotope (^13^C) analysis in combination with molecular markers provides robust tools for the source apportionment of WSOC in marine aerosols^16^.

Three-dimensional excitation emission matrix (EEM) spectroscopy, which yields fluorescence intensities as a function of both excitation and emission wavelengths, has been widely applied to characterize chromophoric dissolved organic matter in terrestrial and oceanic systems^17,18^. However, the method has rarely been used for the analysis of complex organic matter in atmospheric aerosols^19–23^, particularly for marine aerosols. Classifications of organic matter with EEM spectra in SSA need to be employed in comparison with that in SSW, because their sources, chemical compositions, and transformation pathways are expected to be different from those in the marine aqueous environment. In this context, direct comparison of EEMs between SSAs and SSW in the field remains rarely carried out.

Whereas a large fraction of marine organic aerosols (OAs) remains chemically unresolved, a significant fraction of WSOC has been attributed to humic-like substances (HULIS), which are operationally defined as a complex mixture of heterogeneous compounds^24^. Moreover, there is growing evidence that primary producers in the surface ocean may contribute to the humic-like fluorescence in seawater^25^. The major pathways for HULIS production in aerosols include direct emissions (e.g., sea spray, soil resuspension) and secondary formation from both biogenic and anthropogenic precursors (e.g., oxidation, oligomerization, and polymerization). Their formation mechanism and alternative components should be considered, as they may explain the large missing source of marine OA in global models^26^.

Because bioorganic species can affect climate through cloud formation as well as atmospheric chemistry, it is critical to understand the factors that control the release of biomolecules and microbes from the ocean surface to the atmosphere and their roles in aerosol formation. Fundamental nascent chemical properties strongly depend on the geochemical state of source waters at the sea surface. In addition, it is important to understand how the production of SSAs influences the chemical composition of submicrometer and supermicrometer SSAs.

The Oyashio is a cold subarctic ocean current that flows southwestward as a western boundary current of the Western Subarctic Gyre in the North Pacific^27^. In the Oyashio and its coastal region, spring phytoplankton blooming occurs, namely from March to June^28,29^. This oceanic region in spring has the potential for enhanced concentration of OM at the sea surface, which enables us to study the chemical and biological linkages of OM at the ocean–atmosphere boundary.

The objective of this study is to elucidate the chemical transfer of major classes of organic compounds when they move from the bulk SSW to SSAs at the ocean–atmosphere boundary. Based on the shipboard measurements of both marine ambient aerosols and bulk surface seawater in the Oyashio and its coastal region in the western North Pacific (Supplementary Material, Fig. S1), we investigated the chemical transformation of organic compositions during SSW-to-SSA transfer using fluorescence EEM spectroscopy in combination with stable carbon isotopic ratios. Direct comparison of chemical information between SSAs and SSW obtained via EEM analysis enabled us to discuss possible processes of different chemical classes of OM. Our measurements will help to advance the understanding of the underlying processes that determine the distribution and composition of marine aerosols.

## Results and Discussion

### Identification and isolation of the data for sea spray aerosols (SSAs)

The median number concentrations of aerosol particles at dry diameters of 14–710 nm broadly ranged from 497 to 4,947 cm^−3^ for respective periods of the filter samplings with the number of aerosol samples of 22. While these values are similar to those of SSA produced from low temperature (~0–10 °C) seawater using a plunging jet SSA chamber^30^, the result suggests that SSAs could have co-existed with other types of aerosols, on the assumption that the number concentrations of SSAs in pristine marine air are typically <~1,000 cm^−3^ ^31^. Other types of aerosols include particles formed during nucleation events in the open ocean, when their number concentrations can reach ~2,000 cm^−3^ ^32^.

**Figure 1:**
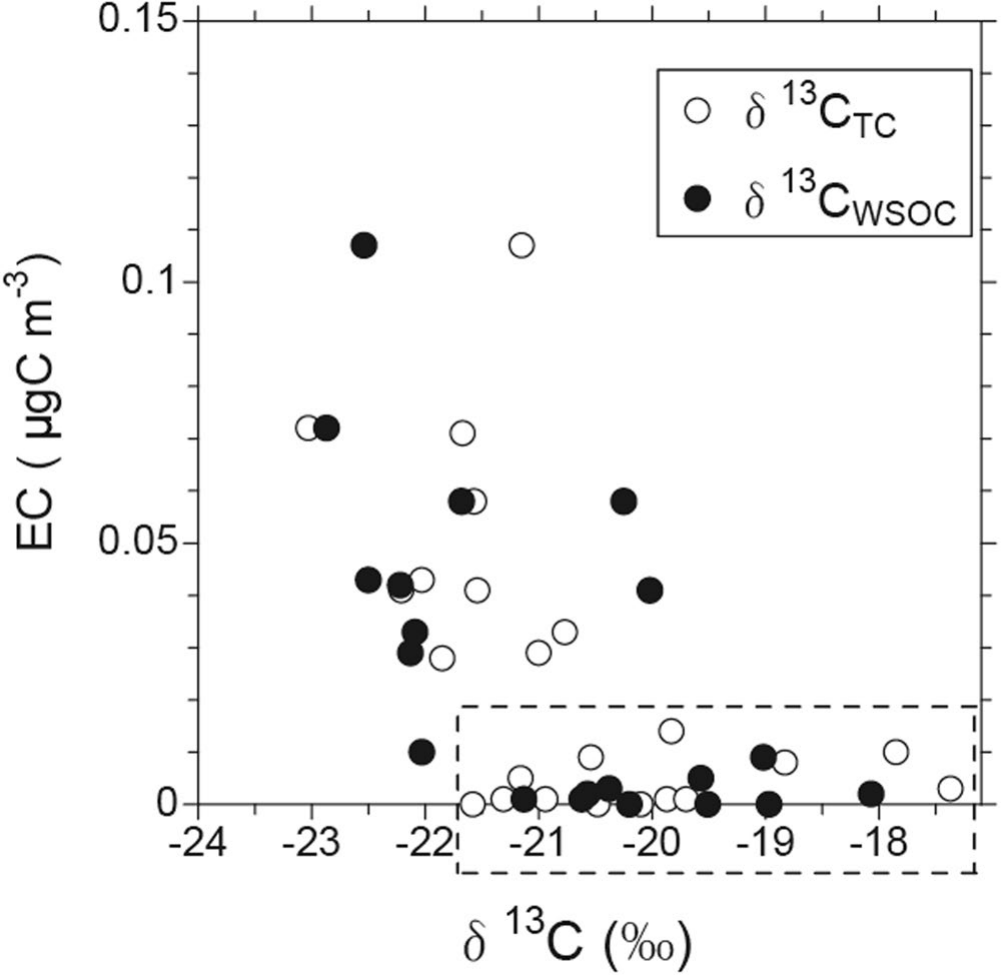
Mass concentrations of elemental carbon (EC) as a function of δ^13^C for total carbon (TC) (δ^13^C_TC_) and water-soluble organic carbon (WSOC) (δ^13^C_WSOC_) in the submicrometer particles collected during the KH-15-1 cruise. A dashed box indicates the range of the data with characteristics of marine sources.

To specifically select the data for the organic component with the characteristics of sea spray, the elemental carbon (EC) concentrations were determined as a function of the stable carbon isotope ratio of WSOC (δ^13^C_WSOC_) in the observed aerosols (Fig. 1). EC is used here as a tracer of terrestrial origin. The δ^13^C_WSOC_ values ranged from −18.1 to −22.9‰ with an average of −20.3 ± 1.7‰. Data with EC concentrations >0.02 µgC m^−3^ showed lower (isotopically lighter) δ^13^C_WSOC_ values, suggesting that these aerosols were influenced by terrestrial sources, mostly anthropogenic ones. In contrast, the data with substantially low concentrations of EC (<0.02 µgC m^−3^) showed the δ^13^C_WSOC_ values generally larger than −22‰. These δ^13^C_WSOC_ values fall within the range of isotopic compositions enriched by marine-derived organic carbon (−22 to −18‰)^16,33^. The same is true for δ^13^C of total carbon (δ^13^C_TC_), whose values were also within the range of marine-derived organic carbon when EC concentrations were below 0.02 µgC m^−3^. Therefore, Fig. 1 clearly demonstrates that δ^13^C can successfully distinguish the contribution between terrestrial and marine origins of WSOC as well as TC in this study. Among the data set indicative of marine origin, six samples showed average local wind speeds of >5 m s^−1^ throughout the aerosol sampling, which were designated sea spray aerosol (SSA) samples in this study. It is noted that isotope equilibrium fractionation linked to photosynthesis of phytoplankton might affect the δ^13^C^33,34^. However, this relation was not distinctly observed between the δ^13^C_WSOC_ in SSA samples and surface sea temperature (SST) during aerosol sampling (data not shown). This is partly attributed to the small variation in the average SST (~0.6–1.4 °C) during SSA sampling in this study.

Table 1 summarizes the major parameters measured in the SSA samples and corresponding SSW samples. The average WSOC/sodium (Na^+^) mass ratio in the SSAs was 1.83 ± 1.30, which is close to the upper end of the OC/Na^+^ ratio range (0.1–2.0) previously reported for submicrometer marine primary OA^35^. If water-insoluble organic carbon (WIOC) is taken into account, the OC/Na^+^ ratio becomes larger than the typical range, probably because the ratio in the coastal region as observed in this study is generally larger than that in the open ocean^36^. The ratio obtained in the current study is consistent with our understanding that the submicrometer SSAs are enriched in OC relative to seawater^4,37^. The concentration of chlorophyll (Chl) *a* is used as a measure of phytoplankton biomass^29^ in the surface ocean. In general, the observed dissolved organic carbon (DOC) concentrations were larger with an increase in Chl *a* levels at the sea surface (Table 1).

**Table 1:** Observed values and ratios of typical parameters in the sea spray aerosols (SSAs) and surface seawater (SSW) sampled during the cruise KH-15-1 in March 2015.

SSA sample ID	SSA sampling date	WSOC (ngC m^−3^)	WSOC/Na^+^ mass ratio	Glucose (ng m^−3^)	MSA (ng m^−3^)	δ^13^C_WSOC_ (‰)	Average local wind speed (m s^−1^)	SSW sample ID	SSW sampling date	DOC (μgC L^−1^)	Chl. *a* (mg m^−3^)
SSA-1	Mar.9	1519	0.638	2.131	143	−20.54	13.8 ± 5.4	SSW-1	Mar.9	756.2	0.374
SSA-2	Mar.13	623	3.839	0.112	21	−21.58	11.5 ± 3.8	SSW-2	Mar.13	803.2	0.756
SSA-3	Mar.14	411	2.907	0.032	51	−19.83	8.5 ± 4.5	SSW-3	Mar.14	809.9	0.755
SSA-4	Mar.17	1486	1.230	1.412	28	−21.03	7.6 ± 3.2	SSW-4	Mar.17	800.4	0.762
SSA-5	Mar.22	805	1.697	0.298	31	−20.77	5.5 ± 1.4	SSW-5	Mar.22	864.6	1.070
SSA-6	Mar.23	969	0.639	0.417	19	−21.85	13.6 ± 1.9	SSW-6	Mar.23	784.1	2.412

**Figure 2:**
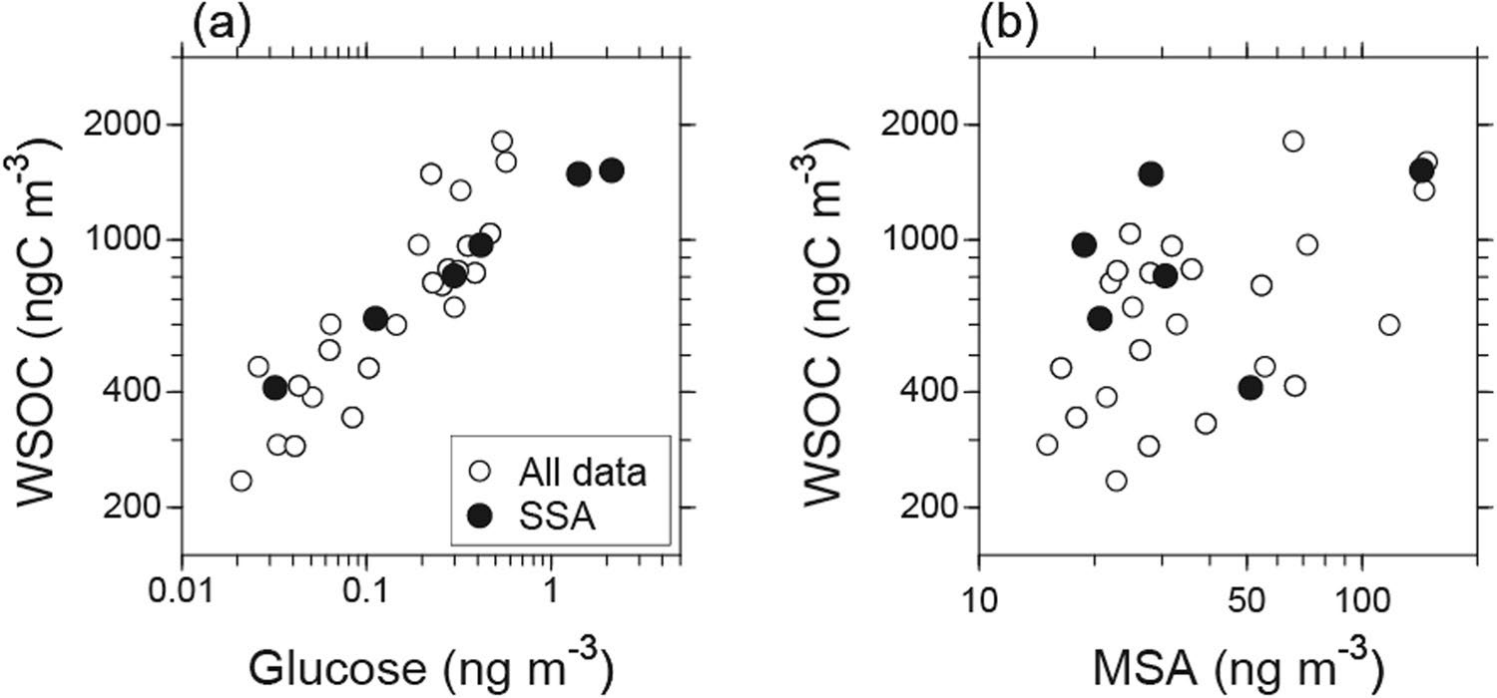
Concentrations of WSOC as a function of (**a**) glucose and (**b**) MSA during the cruise. The solid circles indicate the data of SSAs (i.e., the data with δ^13^C_WSOC_ > −22‰, EC concentrations < 0.02 μgCm^−3^, and the average relative wind speeds >5 m s^−1^).

We also examined organic molecular markers to investigate the contribution of DOC to the submicrometer SSAs defined by the stable carbon isotope ratios. Previous studies in which the sea surface waters were analyzed for organics have revealed a significant carbohydrate concentration^38^. Figure 2a shows the relationship between WSOC and glucose in the SSA and all submicrometer aerosol samples. Here, glucose is used as a tracer for primary marine aerosols, as SSAs have been recognized to contain a substantial amount of monosaccharides, including glucose^16,36^. Indeed, Frossard *et al*.^36^ showed that primary marine aerosol particles generated from bubbled seawater had hydroxyl functional group compositions characteristic of monosaccharides and disaccharides, whereas organics in the seawater had those of polysaccharides, based on Fourier-transform infrared spectroscopy (FTIR) analysis. In our study, the concentrations of WSOC positively correlated with those of glucose, which was particularly evident for SSAs, supporting the nascent characteristic of the SSA samples identified here.

Figure 2b shows a scatter plot of the concentrations of WSOC versus methanesulfonic acid (MSA). An increase in the concentration of MSA did not necessarily accompany the increase in WSOC concentration, particularly for WSOC indicative of SSAs. The observed MSA is considered to be either produced by gas-phase MSA directly scavenged by aerosols or rapidly produced in aqueous phase from scavenged dimethylsulfoxide (DMSO) and methanesulfinic acid (MSIA)^39^, particularly under conditions with high relative humidity typical of the marine boundary layer (MBL). Furthermore, MSA is preferentially produced under low temperatures, such as the average ambient temperature of 2.5 ± 2.3 °C in the study area, due to the temperature-dependent kinetics of MSA formation^40^. Assuming a typical average OH radical concentration of 1 × 10^6^ cm^−3^, a lifetime of DMS is roughly estimated to be <~1 day with respect to oxidation by OH radical in the MBL^41^. The secondary formation of MSA can explain the insignificant correlation with the SSA samples obtained in this study.

### EEM spectra of submicrometer and supermicrometer aerosols vs. bulk seawater

**Figure 3:**
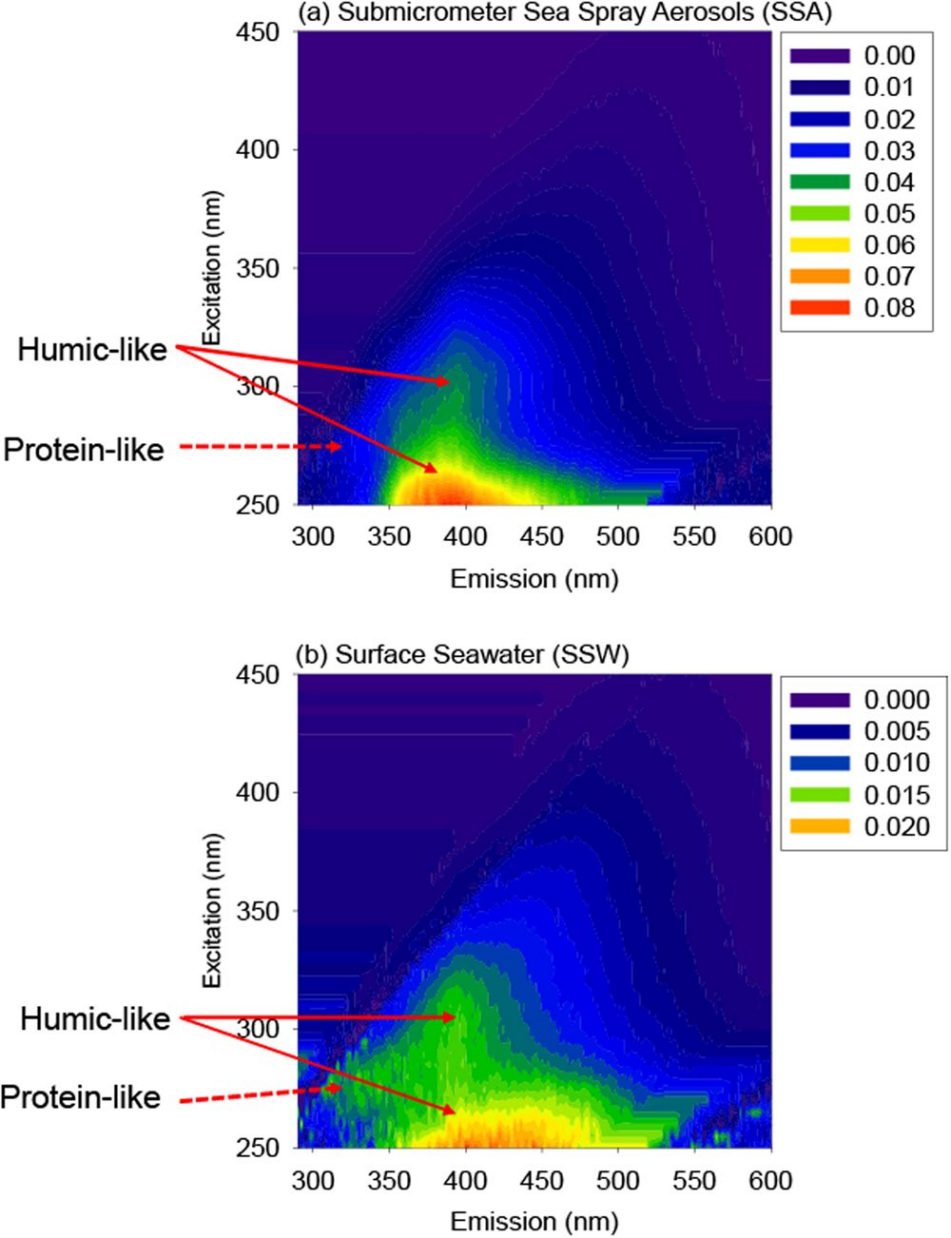
Representative fluorescence excitation-emission matrices (EEMs) for (**a**) the submicrometer SSA samples and (**b**) the bulk surface seawater, obtained on March 9, 2015. Color codes indicate the fluorescence intensity in Raman Units (RU).

Figure 3 presents the EEM spectra of WSOC in the submicrometer SSA samples in comparison with those of DOC in the SSW samples collected on March 9, 2015. The submicrometer SSA and SSW samples displayed a similar distinct peak in the EEM spectrum at wavelengths of excitation/emission (Ex/Em) = 300 nm/400 nm, which is typically defined as peak M^42^. This peak is generally considered of marine humic-like fluorophores^42,43^. It has been reported that HULIS and/or long-chain organics account for a significant fraction of marine aerosols^20,44^. In fact, the aerosol samples other than SSAs also showed distinct peaks of humic-like fluorophores. By using FTIR, Frossard *et al*.^36^ showed a larger fraction of alkane functional groups in SSAs generated from productive seawater than in SSAs generated from nonproductive seawater. Whereas our results corroborate the previous reports of the presence of organic materials with high concentrations observed in both the Pacific and Atlantic Oceans^5,35,45^, these data are significant because they show the consistent prevalence of HULIS and long-chain organics over a wide variety of sampling conditions in the Pacific Ocean.

For the SSW samples, another obvious peak was observed at Ex/Em = 270–280 nm/320–340 nm (peak B and T), which generally corresponds to protein-like fluorophores^42^. These fluorophores are related to marine biological proteinaceous components, including cell or cell fragments, exopolymeric substances, and water-soluble amino acids, peptides, and protein^46,47^. The level of the protein-like component in the seawater was particularly high in the sea surface layers in the onshore during the same cruise, the spatial distribution of which was similar to that of the Chl *a* concentration^48^. For the SSA samples, on the other hand, the fluorescence intensity of the protein-like substances was not apparent in the EEM (Fig. 3a). These results clearly show that the submicrometer SSAs are enriched with humic-like compounds relative to the protein-like compounds, as compared to the SSW.

The biological index (BIX) has been used to estimate the contribution of autochthonous biological activity in surface water^49^. An increase in BIX is associated with an increase in the contribution of microbially derived organics. BIX values larger than 1 have been shown to correspond to DOM of aquatic microbial origin^49^, whereas lower values (e.g., <0.5) indicate contribution of soil origin^50^. The BIX values for the SSAs in the present study (0.74–1.15 with an average of 0.88 ± 0.18) (Table 2) correspond well to the typical ranges of microbial-origin DOM. Indeed, the BIX for the SSW ranged between 0.99 and 1.14, with an average of 1.09 ± 0.05. These results suggest that the majority of the DOM sampled was biologically derived, which significantly contributed to the SSAs observed in this study.

**Table 2:** Fluorescence spectral parameters of the SSA and SSW samples.

SSA sample ID	Protein-like (Ex/Em = 275/330 nm)	Humic-like (Ex/Em = 300/400 nm)	Humic-like/Protein-like	BIX (Ex = 310 nm, Em = 380/430 nm)	SSW sample ID	Protein-like (Ex/Em = 275/330 nm)	Humic-like (Ex/Em = 300/400 nm)	Humic-like/Protein-like	BIX (Ex = 310 nm, Em = 380/430 nm)
SSA-1	0.0264	0.0367	1.392	1.13	SSW-1	0.0118	0.0133	1.130	1.08
SSA-2	0.0212	0.0464	2.190	0.76	SSW-2	0.0138	0.0160	1.159	1.05
SSA-3	0.0269	0.0529	1.967	0.78	SSW-3	0.0144	0.0211	1.465	0.99
SSA-4	0.0840	0.2207	2.626	0.81	SSW-4	0.0142	0.0147	1.036	1.08
SSA-5	0.0222	0.0477	2.143	0.74	SSW-5	0.0258	0.0135	0.523	1.14
SSA-6	0.1119	0.1474	1.317	1.15	SSW-6	0.0135	0.0130	0.961	1.13

The fluorescence is in Raman Units (RU; nm^−1^).

**Figure 4:**
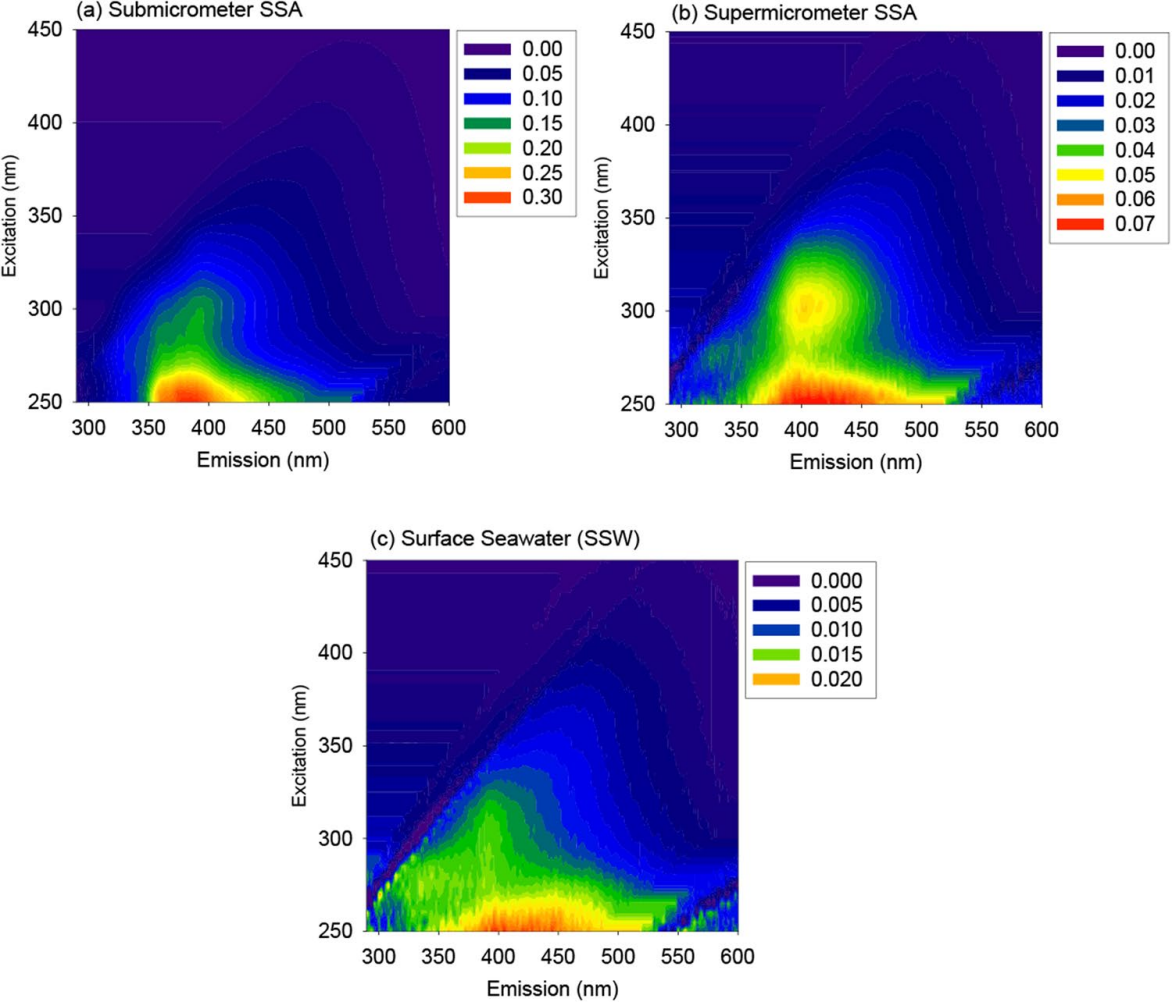
EEMs for the (**a**) the submicrometer SSAs, (**b**) supermicrometer SSAs, and (**b**) bulk surface seawater obtained on March 23, 2015. Note that the scales of color-coded EEM intensities in RU are different for each panel.

To investigate the dependence of the EEM spectra on the aerosol particle size, the spectra of WSOC in submicrometer and supermicrometer SSAs were compared with that of SSW collected on March 23, 2015 (Fig. 4). The EEM spectra of WSOC in the supermicrometer SSA were similar to that of the submicrometer SSA. The similarity in the EEM spectra supports the idea that the majority of protein-like compounds were not enriched in the supermicrometer size mode of SSAs, but rather preferentially remained in the SSW during the SSA production or were decomposed during the process of sea-to-air transfer. To summarize, these results suggest substantial transformation in composition of DOM during transfer from the SSW to SSAs, regardless of the aerosol particle size.

### Implications for the sea-to-air transfer of dissolved organic matter

**Figure 5:**
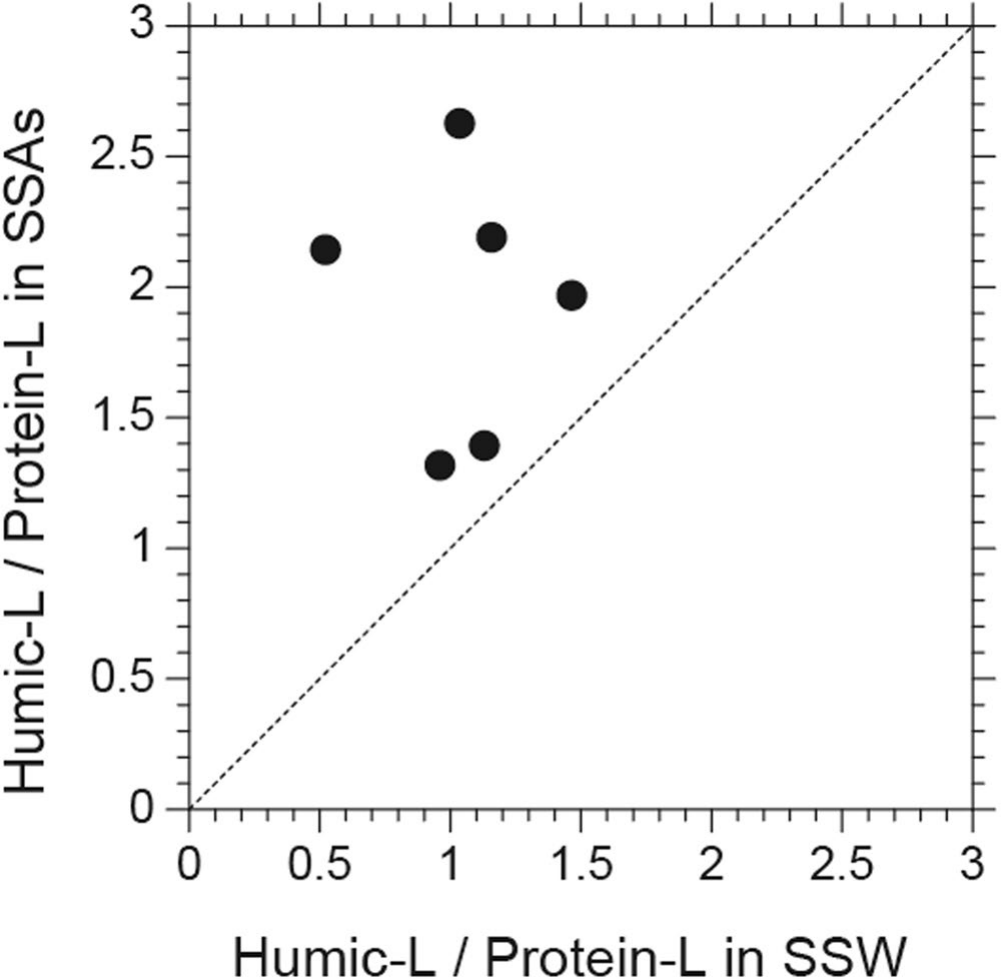
A scatterplot of the fluorescence intensity ratios of humic-like (L)/protein-like (L) compounds between the submicrometer SSA and the SSW samples. A dashed line indicates a 1:1 line.

Figure 5 shows a scatterplot of the fluorescence intensity ratios of humic-like/protein-like compounds between the submicrometer SSAs and the SSW samples during the entire cruise. In spite of the limited number of the data, all the ratios for the SSAs were larger (by 63% in median) than those in the corresponding SSW samples. The higher ratios of the humic-like/protein-like fluorescence intensity in aerosols can be attributed to (i) the retention of protein-like DOM in the SSW, (ii) photochemical or biological depletion of protein-like compounds relative to humic-like compounds in the SSAs, and/or (iii) efficient formation of humic-like substances in the SSAs. These processes possibly occurred within the timescale of the aerosol sampling (~12–24 h).

The retention of protein-like DOC relative to humic-like compounds in the SSW is likely the result of the protein-like DOC not being incorporated into the bubble films that produce sea spray particles. This preferential partitioning is attributable to proteins associated with exopolymer gels in SSW^46^, in which colloidal structures can be prevented from being incorporated into the bubble films to form SSAs^51^. In addition, if protein-like DOC formed a smaller amount of large particulate matter in SSW than did humic-like DOC, it would be less incorporated into SSAs relative to humic-like DOC.

Another possible explanation with regard to (ii) and (iii) is that protein-like compounds could be degraded via ultraviolet radiation and/or microbial activity at the sea surface, which reduced the abundance of protein-like DOC at the sea surface. Yamashita and Tanoue^18^ conducted a degradation experiment using natural phytoplankton to investigate temporal changes in marine humic- and tryptophan-like compound (one of the protein-like compounds) fluorescence intensities in artificial seawater samples. They demonstrated that marine humic-like fluorophores were rapidly produced on a timescale of a day, whereas tryptophan-like fluorophores from natural phytoplankton were rapidly degraded. That study supports that proteins can be converted to processed oligomers^46^ at the air–sea interface on timescale considered here, which is likely associated with microbial production of HULIS. The preferential formation of HULIS in the SSAs found in the current study can be explained by aggregation of the precursor materials of HULIS^24,52^, which include the degraded materials of protein-like compounds.

In this work, the observed fluorescent component in the SSAs with a spectral location at around Ex/Em = 300/400 nm was attributed to biologically derived HULIS. This indicates that selective formation and/or transition of HULIS during SSA production from DOC at the ocean surface can potentially play a significant role in determining the chemical and physical properties of aerosol particles in the MBL. Overall, this study demonstrated that the changes in chemical composition occur as freshly injected DOC into the atmosphere, which is chemically and/or biologically modified on a timescale of a half to one day. Future studies are required to conduct similar measurements of the linkage of chemical characteristics at the air–sea interface during phytoplankton blooms and post-blooms. Additional measurements are needed to elucidate the mechanism and relative contributions of photochemical and biological effects on the enrichment of HULIS rather than of protein-like substances compared to those in seawater. This study provides unique insights into the complex transfer of biologically derived organic species from the ocean surface to the atmosphere.

## Methods

### Ambient aerosol sample collection

Ambient submicrometer aerosol samples were collected from March 6–25, 2015, on board the R/V *Hakuho Maru*. The sampling was carried out during cruise KH-15–1 in the Oyashio and its coastal regions in the western North Pacific (Supplementary Material, Fig. S1)^48^. The aerosol samples were collected continuously using a high-volume air sampler (HVAS; Model 120SL, Kimoto Electric, Osaka, Japan) on the deck above the bridge of the ship. A cascade impactor (CI; Model TE-234, Tisch Environmental, Cleves, OH, USA) attached to the HVAS was used to collect size-segregated particles^16^ at a flow rate of 1130 L min^−1^ without temperature and humidity control. In this study, we used analytical results obtained from the bottom and upper stages of the impactor, which collected particles with aerodynamic diameter (D_p_) < 0.95 μm and D_p_ > 0.95 μm, respectively. In the present study, aerosol particles collected at the bottom and upper stages were referred to as submicrometer and supermicrometer particles, respectively.

The aerosol sampling was conducted during local daytime and nighttime with the duration of each sampling approximately 12–24 hours. The samples were collected on quartz fiber filters (25 cm × 20 cm), which were pre-combusted at 410 °C for 6 hours to remove any contaminants. Collected filters were individually stored in glass jars with a Teflon-lined screwed cap at −20 °C to limit chemical reactions on the filter and losses of volatile compounds. Possible contamination from the ship exhaust was avoided by shutting off the sampling pump when air came from the beam and/or when the relative wind speed was low (<5m s^−1^). As a result of the data screening, we present 22 samples of the submicrometer particles for the data analysis out of the total 33 samples obtained in the present study.

### Collection and analysis of bulk surface seawater (SSW) samples

SSW was collected using a bucket during each aerosol sampling period during the same cruise (Table S1). Average temperature of SSW at each time of the collection was 1.0 ± 0.6 °C. Further details of SSW properties during the cruise are given by Mizuno *et al*.^48^ The seawater samples were filtered with a 0.22-μm Durapore filter (Millipak, Millipore) under gentle vacuum. The filtrate was transferred into a pre-combusted glass vial with an acid-cleaned Teflon-lined cap^48^. The samples were then stored frozen at −20 °C in the dark until analysis.

For Chl. *a* measurement, duplicate seawater samples (each 0.5–1.2 L) were collected at 0 m of each sampling station using a clean plastic bucket, and filtered into 25 mm Whatman GF/F filters (nominal pore size of 0.7 µm) with gentle vacuum (<0.013 MPa). Each filter was blotted with a filter paper, frozen in liquid nitrogen, and then stored in an ultra-freezer (<−70 °C) until analysis on land. Following Suzuki *et al*.^53^, phytoplankton pigments including Chl. *a* were extracted with a DMF-bead-beating technique and analyzed with ultra-high performance liquid chromatography (UHPLC). In addition, DOC concentrations were measured according to Tanaka *et al*.^54^ Briefly, samples for DOC analysis were filtered with a 0.22-μm Durapore filter (Millipak, Millipore) under gentle vacuum and frozen (−20 °C) immediately after collection to store in precombusted borosilicate glass vials. DOC analysis was conducted by using a total organic carbon (TOC) analyzer (Model TOC-V, Shimadzu). Six SSW samples are presented for the data analysis out of the total 14 samples in this study.

### Stable carbon isotopic characterization of water-soluble organic aerosols

For the determination of δ^13^C_WSOC_, a filter (14.14 cm^2^) for each sample was acidified to pH 2 with hydrochloric acid (HCl) to remove inorganic carbon prior to extraction. The decarbonated filter samples were then dried under a nitrogen stream for approximately 2 hours. WSOC was extracted from the filters in 20 mL of ultrapure water using the method described above for measuring the WSOC concentration. The extracted samples were concentrated via rotary evaporation, and 40 μL of each sample was transferred to be absorbed onto 10 mg of pre-combusted Chromosorb in a pre-cleaned tin cup. The ^13^C_WSOC_ was then measured using an elemental analyzer (EA; NA 1500, Carlo Erba, Milan, Italy) interfaced with an isotope ratio mass spectrometer (IRMS; Finnigan MAT Delta Plus, Thermo Finnigan, San Jose, CA, USA). In addition, the δ^13^C of total carbon (δ^13^C_TC_) (i.e., without water extraction) was also measured with the EA–IRMS for the same aerosol filter samples. The ^13^C data were reported relative to an established reference of carbon Vienna Pee Dee Belemnite (VPDB). Further details of the analytical method used for isotopic analysis are given by Miyazaki *et al*.^16^.

### Excitation fluorescence characterization

Fluorescence characteristics were obtained for both submicrometer/supermicrometer water-soluble aerosol and surface seawater samples. For the aerosol samples, a filter cut of 19.63 cm^2^ was extracted using 20 mL ultrapure water in an ultrasonic bath (5 min × 3). The extracts were filtered through a 0.22- µm pore syringe filter (Millex-GV, Millipore) to remove any insoluble particles with diameters larger than 0.22 µm.

Excitation-emission matrix (EEM) fluorescence was measured using a fluorometer (FluoroMax-4, Horiba)^48,54^. All fluorescence spectra were acquired in the S/R mode with instrumental bias correction. After this procedure, the EEM of Milli-Q water was subtracted from the sample EEMs. Finally, each EEM was calibrated to the water Raman signal, and the fluorescence is reported in Raman Units (RU; nm^−1^). Based on the ranges of Coble^42^, the fluorescence intensities of peak M (humic-like, Ex/Em = 300/400 nm) and T (protein-like, Ex/Em = 275/330 nm) were obtained from excitation/emission pairs. The biological index (BIX) is defined as the ratio of emission intensities at Em = 380 nm and 430 nm with excitation at Ex = 310 nm calculated from the EEM^50^.

It is noted that the EEM signal likely contains the effect of charge transfer processes^55^. This effect is likely insignificant in this study, because we discuss the relative intensities of the fluorescence of humic-like and protein-like compounds between SSAs and SSW samples. Furthermore, large responses of fluorescence to pH are known to be severe at extremes of pH^56^. However, within natural levels, typically observed in seawater as well as aerosol samples extracted with ultrapure water (almost neutral), the response to pH is considered to be insignificant^57^ in this study.

### Chemical analysis of water-soluble aerosols

The term water-soluble aerosols in the present study is technically defined as particles sampled on the filter and extracted with ultrapure water followed by filtration through a syringe filter^58^. To determine the WSOC concentration of the submicrometer filter samples, another filter cut of 3.14 cm^2^ was extracted with 20 mL ultrapure water using an ultrasonic bath for 15 min. The extracts were filtered through the same type of 0.22-µm pore syringe filter as described above, before being injected into a TOC analyzer (Model TOC-L_CHP_, Shimadzu)^59^.

Additionally, another cut of the filter (3.14 cm^2^) was extracted with 10 mL of ultrapure water under ultrasonication to determine the concentration of major inorganic ions, including Na^+^. The same syringe filter type as described above was used, before the extract was injected into an ion chromatograph (Model 761 compact IC; Metrohm)^59^. Mass concentrations of elemental carbon (EC) were measured using a Sunset Laboratory OC-EC analyzer. A filter punch of 1.54 cm^2^ was used for this analysis.

Another portion of the filter (3.80 cm^2^) was extracted with dichloromethane/methanol to measure glucose as a biogenic molecular tracer. The –OH functional group in the extracts was reacted with N,O-bis-(trimethylsilyl) trifluoroacetamide (BSTFA) to form trimethylsilyl (TMS) ethers. The TMS derivatives were then analyzed using a capillary gas chromatograph (GC7890, Agilent) coupled to a mass spectrometer (MSD5975C, Agilent)^59^. The chemical measurement data are presented in Table 1.

### Aerosol number concentrations

For measurement of aerosol number concentrations, ambient aerosol was aspirated at a flow rate of 17 L min^−1^ on the upper deck of the ship. The inlet was placed at the front edge of the upper deck facing the bow to avoid sampling of the ship exhausts. The aerosol was drawn to the laboratory beneath the deck, passed through the PM_2.5_ cyclone. It was then introduced to a scanning mobility particle sizer (SMPS), which is composed of a differential mobility analyzer (Models 3080 and 3081, TSI Inc.) and a condenstion particle counter (Model 3775, TSI Inc.). The data were corrected by taking account of diffusion loss of particles in the sampling line and the SMPS^60^. To remove data that were influenced by the ship exhaust, data obtained when the relative wind direction was within 60° from the bow and when relative wind speed was ≥5 m s^−1^ were used.

## Electronic supplementary material

Supplementary Information
