# Supplementary material for: Chemical transfer of dissolved organic matter from surface seawater to sea spray water-soluble organic aerosol in the marine atmosphere
Source: Sci Rep. 2018 Oct 5;8:14861. doi: 10.1038/s41598-018-32864-7 (PMC6173719; doi:10.1038/s41598-018-32864-7)
Supplement: Supplementary file 1 — Supplementary Information [file 41598_2018_32864_MOESM1_ESM.docx]

**Supplementary Information for the manuscript:**

**Chemical** **transfer of dissolved organic matter from surface seawater to sea spray water-soluble organic aerosol in the marine atmosphere**

Yuzo Miyazaki^1^, Youhei Yamashita^2^, Kaori Kawana^3,4^, Eri Tachibana^1^, Sara Kagami^3^, Michihiro Mochida^3,5^, Koji Suzuki^2^ & Jun Nishioka^1^

^1^Institute of Low Temperature Science, Hokkaido University, Sapporo, 060-0819, Japan

^2^Faculty of Environmental Earth Science, Hokkaido University, Sapporo, 060-0810, Japan

^3^Graduate School of Environmental Studies, Nagoya University, Nagoya, 464-8601, Japan

^4^Now at School of Materials and Chemical Technology, Tokyo Institute of Technology, Tokyo, 152-8550, Japan

^5^Now at Institute for Space–Earth Environmental Research, Nagoya University, Nagoya, 464-8601, Japan

Correspondence and requests for materials should be addressed to Y. M. (email: yuzom@lowtem.hokudai.ac.jp)


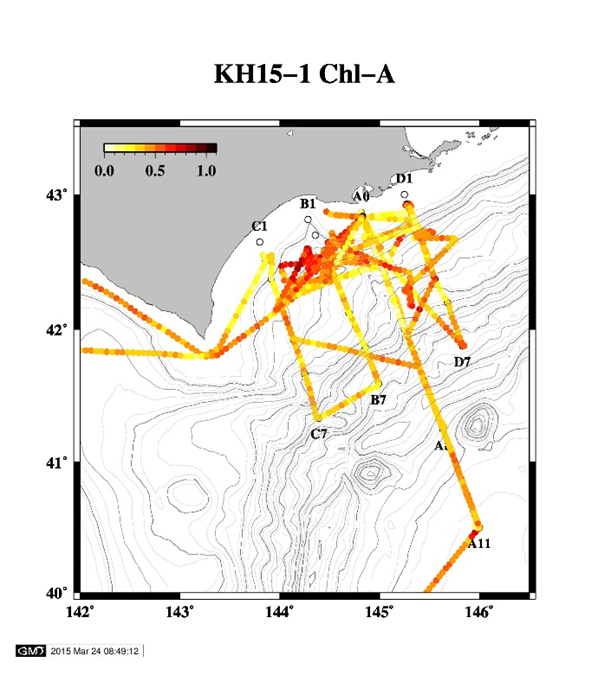

**Figure S1.** KH-15-1 cruise track shown in red (left) and relative in vivo Chl *a* fluorescence levels at ca. 5 m along the track (right). The fluorescence levels were determined with a Chl *a* fluorometer (Model FLRT, WET Labs Inc.) of the surface monitoring system.

**Table S1**. Location of the sampling points of surface seawater (SSW) during the cruise KH-15-1 in March 2015.

| SSW sample ID | Sampling date | Longitude (ºE) | Latitude (ºN) |
| --- | --- | --- | --- |
| SSW-1 | Mar.9 | 144.91 | 42.69 |
| SSW-2 | Mar.13 | 144.50 | 42.50 |
| SSW-3 | Mar.14 | 144.48 | 42.49 |
| SSW-4 | Mar.17 | 145.01 | 42.55 |
| SSW-5 | Mar.22 | 144.40 | 42.53 |
| SSW-6 | Mar.23 | 145.26 | 42.92 |
